# Isolation, Cloning and High- Level Expression of Neutrophil Gelatinase-Associated Lipocalin Lipocalin2 by Baculovirus Expression System through Gateway Technology

**Published:** 2012

**Authors:** Mahdi Rouhbakhsh, Raheleh Halabian, Nasser Masroori, Mahshid Mohammadi Pour, Parisa Bahmani, Amaneh Mohammadi Roush, Ali Jahanian-Najafabadi, Mehryar Habibi Roudkenar

**Affiliations:** 1 *Blood Transfusion Research Centre, High Institute for Research and Education in Transfusion Medicine, Tehran, Iran *; 2 *Department of Anatomy, Faculty of Medicine, Medical University of Hamadan, Hamadan, Iran*; 3 *Department of Molecular Biology, Pasteur Institute of Iran, Tehran, Iran*

**Keywords:** Baculoviridae, Lipocalin 2 (Lcn2), Neutrophil gelatinase-associated lipocalin (NGAL), Recombination, Vector

## Abstract

**Objective(s):**

Lipocalin 2 (Lcn2) is a 25-kDa glycoprotein that has initially been extracted from neutrophil granules. Expression of Lcn2 is induced under various pathophysiological conditions. It is also known as an early marker of kidney and heart injury. High-level expression of recombinant Lcn2 neutrophil gelatinase-associated (NGAL) in insect cells was the aim of this study.

**Materials and Methods:**

Lcn2 gene was isolated from HepG2 cell line. The PCR product was cloned into TOPO vector to construct TOPO-Lcn2. Then Lcn2 was transferred to Gateway adapted Baculovirus DNA by LR recombination reaction. The recombinant Baculovirus DNA was transfected** i**nto insect cell line. Expression of recombinant Lcn2 was detected by RT-PCR, ELISA and western blot analysis.

**Results:**

Insertion of Lcn2 into pENTR/D-TOPO vector was confirmed by using PCR. The accuracy of the nucleotides sequence was verified by DNA sequencing. Transfer of the Lcn2 cDNA into the Baculovirus destination vector by LR recombination reaction was confirmed by amplification of a band of about 860 bp length by using forward Lcn2 primer and V5 reverse primer. Next, Lcn2 protein was detected as a prominent band with approximate molecular weight of 30 kDa in SDS-PAGE and western blot analysis. ELISA results revealed high-level expression of Lcn2 by *Spodoptera frugiperda* (Sf9) cells.

**Conclusion:**

High-level expression of Lcn2 protein in insect cells is promising for future potential application**s**. Recombinant Lcn2 might be used for producing monoclonal or polyclonal antibodies and as potential therapeutic agent. Large scale expression and purification are next steps that are on the way.

## Introduction

 The Lipocalin protein family contains more than 20 small secreted proteins that generally bind soluble extra cellular macromolecules, small hydrophobic ligands, and may not be some specific cell surface receptors (1, 2). The Lipocalins comprise a class of proteins that are characterized by highly conserved beta sheet structure, forming a beta-barrel defining a calyx (3). The neutrophil gelatinase-associated lipocalin (NGAL) or lipocalin2 (Lcn2) is a member of the lipocalin super family with diverse functions such as suppression of bacterial growth, cytoprotection against ROS, and modulation of inflammatory responses (4, 5). Moreover, it has been shown that Lcn2 is an adaptive response to ameliorate the injuries induced by thermal stresses for reestablishment of homeostasis (6).

Lcn2 is known as an acute-phase protein that can protect the body against acute ischemic renal injury, therefore it is suggested that administration of exogenous Lcn2 may exerts remarkable protection in AKI (Acute Kidney Injury ) (7, 8).

In addition, Lcn2 exerts bacteriostatic effects by capturing and depleting the siderophores. This creates a role for Lcn2 in tissue-protection against infection (9, 10). Furthermore, the application of recombinant Lcn2 as a therapeutic agent can be one of the potential solutions for some diseases such as ischemic renal injury and infection. With regard to some of these functions, this protein has been known as a candidate for medical application. Therefore, cloning and expression of Lcn2 could be of great importance in medicine. The expression of Lcn2 gene has been reported in prokaryotic and mammalian expression systems. In prokaryotic system, human NGAL has been expressed in *Escherichia coli* strain BL21 fused with N-terminal OmpA signal peptide and C-terminal Strep-tag II from the vector phNGAL14 (11). In the case of its expression in mammalian cells, the NGAL cDNA has been cloned into the pGEM-T-easy plasmid, followed by digestion with restriction endonucleases and cloned into the corresponding sites in the pLenti6/V5 viral vector. Then, the cell culture medium containing the recombinant viruses has been used to transduce KM12SM cells (12). In other studies, the amplified FLAG-tagged NGAL cDNA has been subcloned into the mammalian expression vector pcDNA3.1/HisC and transfected to A549 and CHO cells (13,14), but as far as we know, NGAL has not been expressed in baculovirus expression system before. Furthermore, in those studies traditional strategies (excision with restriction enzymes and ligation with DNA ligase) have been used for cloning of the Lcn2 gene. 

In the present study gateway technology and baculovirus expression system were used to provide a rapid and highly efficient way for high level expression of recombinant Lcn2. It is noteworthy that in contrast to traditional cloning methods, gateway technology does not require digestion with restriction endonucleases and ligase mediated joining of the fragments. In the present study, we transiently overexpressed the human Lcn2 gene in Sf9 cells using baculovirus expression system.

## Materials and Methods

***Isolation and cloning of Lcn2 coding sequence***

Initially, total RNA of HepG2 cells was extracted by Sinagen RNA extraction kit according to the manufacturer’s instructions (Sigma, USA). Then, 1 µg of the total RNA was used to generate the first-strand cDNA using the cDNA synthesis kit (Invitrogen, USA) and random hexamers as primers. The RT-PCR reaction condition included 25 ^o^C for 10 min, 50 ^o^C for 50 min, and 85 ^o^C for 5 min. 

Isolation of full length Lcn2 cDNA was performed with Pfu DNA polymerase in GeneAmp PCR system 9600 thermocycler (PerkinElmer Life and Analytical Sciences, Wellesley, MA).The following primers were used for amplification of the sequence: 5’-CACC ACG AAT TCA CCA TGG TGC CCC TAG GTC TCC TGT GGC TG-3’ as forward and 5’-TAG CGG CCG CTC AGC CGT CGA TAC ACT GGT C-3’ as reverse. The underlined sequence was introduced to the forward primer for TOPO cloning as mentioned by the manufacturer. PCR was performed under standard condition and finally, gel electrophoresis was performed for further analysis of the PCR products.

The cloning of Lcn2 in pENTR/D-TOPO vector was carried out by pENTR directional TOPO cloning kit (Invitrogen, USA). Briefly, 20 ng of the cDNA and 20 ng pENTR/D-TOPO DNA were mixed and followed by 5 min incubation at room temperature. Finally 2 µl of the reaction were used to transform chemically competent *E. coli* cells. The recombinant bacteria were screened using LB agar medium (Merck, Germany (containing 50 μg/ml kanamycin. The recombinant plasmid pENTR-Lcn2 was extracted from positive colonies using High Pure Plasmid Isolation Kit (Roche, Germany) and then analyzed for the proper orientation of the cloned fragment by PCR and DNA sequencing (Accession Number EU644752)

***Generation of recombinant baculoviruses carrying Lcn2***

Two hundred ng of the recombinant entry plasmid (pENTR-Lcn2) extracted from positive colonies and 300 ng of linear baculovirus destination vector were mixed according to the manufacturers’ instructions (Invitrogen), and then incubated at 25 ^o^C for 1hr. The accuracy of the recombination reaction was analyzed by PCR using polyhedrin forward primer (AAATGATAACCATCTCGC) and V5 reverse primer (Invitrogen).

***Cell culture***

HepG2 cell line, obtained from the National Cell Bank of Iran, was cultured in RPMI-1640 medium (Gibco-BRL, Eggenstien, Germany) containing 10% fetal bovine serum (FBS), 1 µg/ml vitamin K1, 100 U/ml penicillin, and 100 µg/ml streptomycin (Gibco).* Spodoptera frugiperda*, Sf9 cells (Invitrogen) were grown in complete TNM-FH containing supplemented grace’s insect medium (Invitrogen), 10% FBS (Gibco) and penicilin-streptomycin (100 µg/ml) and incubated at 27 ^o^C without CO_2_ exchange.

***Transfection of SF9 cells***

The recombinant baculovirus carrying Lcn2 was transfected into Sf9 cells using Cellfectin reagent as described by the manufacturer (Invitrogen). The supernatant containing recombinant budded viruses was harvested 96 hr post-infection and centrifuged at 4000 rpm for 5 min to remove cell debris. The obtained viruses were amplified through three consecutive rounds of Sf9 cells infection. 

***Expression of recombinant Lcn2***

Expression of Lcn2 was performed by infection of approximately 810^5^ Sf9 cells using the third generation (P3) of the recombinant viruses. Sf9 cells were transfected at high multiplicity of infection (MOI, 20 plaques forming units per cell) and the cellular and medium fractions of transfected cells were harvested in different time intervals (48, 72, 96 and 120 hr). To assess the accuracy of the Lcn2 expression, RT-PCR was performed for the medium fractions of 48 and 72 hr, against the untransfected cells as negative control. 

***Analysis of the recombinant Lcn2***

In order to evaluate the expression of the Lcn2 protein, SDS-PAGE and western blot analysis were performed as previously described (14). The SDS-PAGE gel was stained with an enhanced Coomassie blue dye reagent. The western blot analysis was performed through using PVDF membrane. Immune detection of Lcn2 was carried out using the primary goat anti human Lcn2 antibody and a secondary HRP conjugated anti goat antibody. Then the reaction was developed by ECL kit (Amersham, USA). 

Expression of Lcn2 was also investigated by sandwich ELISA method (R&D Systems, Minneapolis, MN) in triplicate for all fractions, as instructed by the manufacturer and by utilization of the peroxidase coupled rabbit anti-mouse IgG (Dako, Glostrup, Denmark) .

## Results

***Isolation of Lcn2 gene and generation of the recombinant viral vector***

**Figure 1:**
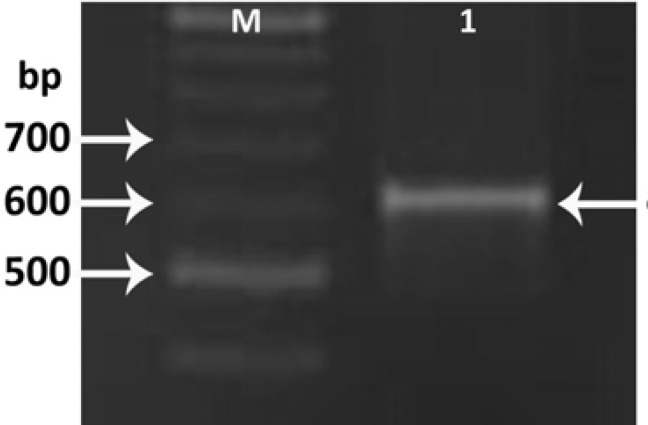
Isolation of the Lcn2 cDNA by specific primers from cell line (lane 1), single 625 bp band was confirmed. M; 100 bp ladder molecular weight marker

Isolation of Lcn2 cDNA was performed by PCR using specific primers following the synthesis of total cell cDNAs by RT-PCR. PCR was performed by pfu DNA polymerase and the expected band of about 625 bp was observed on agarose gel electrophoresis (Figure 1). 

**Figure 2:**
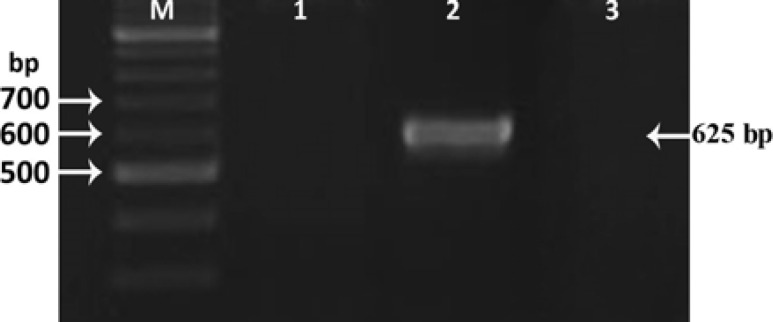
PCR was performed to confirm the correct insertion of Lcn2 into the appropriate entry vector (pENTR-TOPO). Three clones were analyzed by PCR using the specific primers for Lcn2. Results confirmed the second clone to be recombinant. M; 100 bp DNA marker

**Figure 3:**
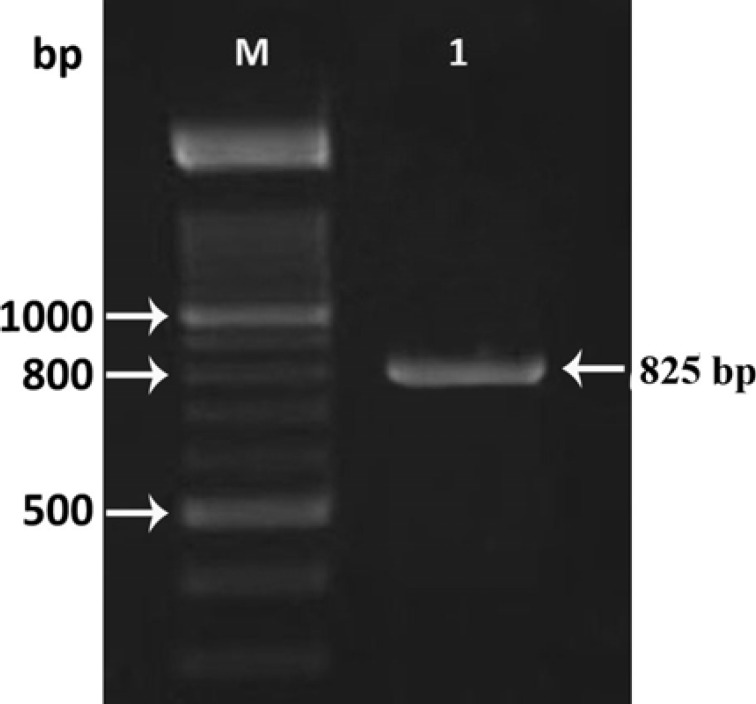
PCR was performed to confirm the correct insertion of Lcn2 into the appropriate bacmids. Transposition of Lcn2 into the bacmid supplied by DH10Bac cells was confirmed by PCR using M13 primers, which resulted in a single 800 bp band. M; 100 bp DNA marker

PCR reaction using the Lcn2 specific primers was also performed to evaluate the recombinant clones and to confirm the direction of the cloned gene within the pENTR/D-TOPO plasmid (Figure 2). For further evaluation, the PCR reaction was performed using M13 primers to amplify an 825 bp-fragment (Fig. 3) confirming the insertion of the Lcn2 gene into the pENTR/D-TOPO plasmid. Finally, the accuracy of the nucleotides sequence was confirmed by DNA sequencing.

***Generation of recombinant baculoviruses***

**Figure 4:**
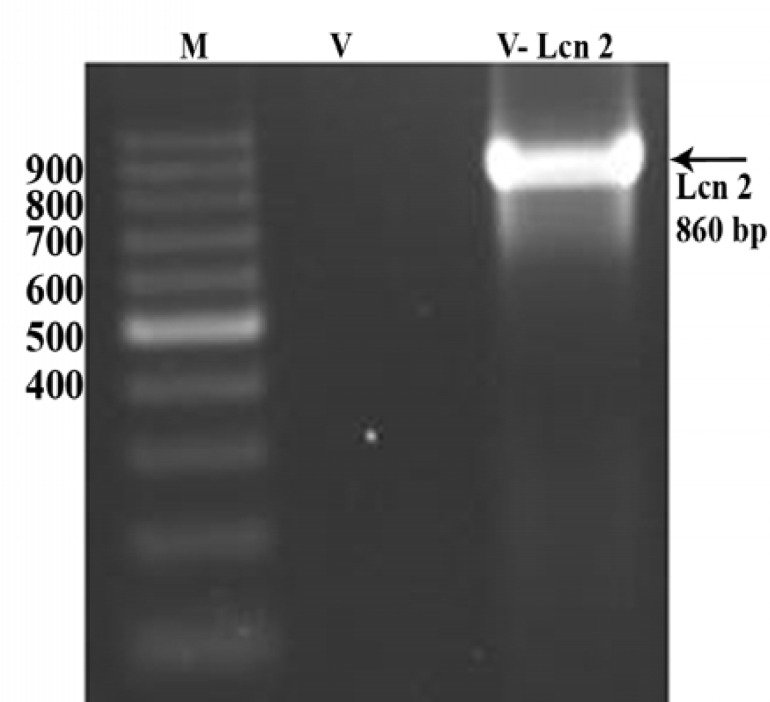
PCR was performed to confirm the correct insertion of Lcn2 into the appropriate bacmids. The accuracy of recombination reaction was determined through PCR by using polyhedron forward and V5 reverse primers and resulted in a band of about 860 bp. M; 100 bp ladder marker.

PCR reaction was performed using polyhedrin forward primer and V5 reverse primer to confirm the transfer of the Lcn2 cDNA into the baculovirus destination vector by LR recombination reaction. This resulted in amplification of a band of about 860 bp length (Figure 4).

***Evaluation of the Lcn2*** ***expression***

**Figure 5:**
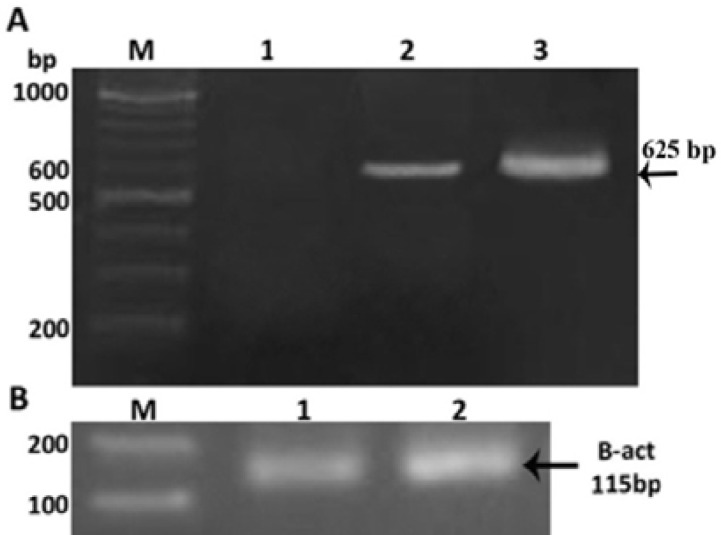
Expression of Lcn2 by transfected Sf9 cells RNA was extracted from Sf9 cells, cDNA was synthesized, and RT–PCR was performed. A: The presence of a Lcn2 mRNAs in Sf9 cells transfected with the recombinant baculoviruses was confirmed by RT–PCR after 48 hr (lane 2) and increased over time on 72 hr (lane 3). No expression was observed in non-transfected Sf9 cells (lane 1). B: The expression of beta actin in both non-transfected SF9 cells and Sf9 cells transfected with recombinant viruses. M; 100 bp ladder marker.

**Figure 6:**
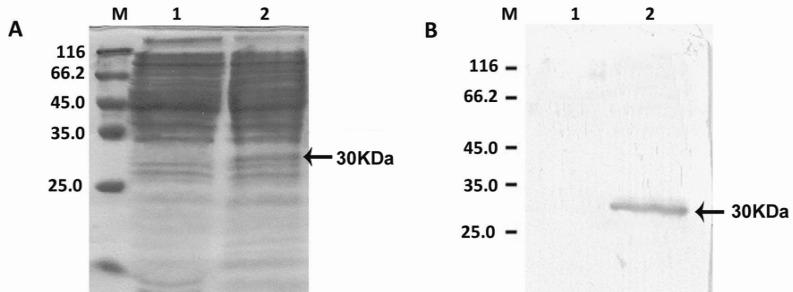
A: SDS-PAGE and B: Western blot analysis of Lcn2 expression. Total protein of Sf9 cell lysates transfected with the recombinant virus was extracted. Existence of a band of about 30 kDa indicated the expression of recombinant Lcn2 (lane 2). M is the protein molecular weight marker

The presence of Lcn2 mRNA transcript in Sf9 cells was confirmed by RT-PCR and the results showed that the expression of the recombinant protein increased over time (Figure 5). SDS-PAGE and western-blot analysises were performed for evaluation of the recombinant protein production using specific Lcn2 antibody (Figure 6). The human Lcn2 migrated as a prominent band with approximate molecular weight of 30 kDa in SDS-PAGE. The results indicated the expression of recombinant Lcn2 with an approximate molecular weight increasing from 30 kDa that is due to the production of recombinant protein. 

Expression of human Lcn2 by Sf9 cells was further determined by ELISA. Results showed that the cells transfected with Lcn2 express Lcn2 protein, whereas cells transfected with PENTR vector do not (Table 1).

**Table 1:** ELISA for human Lcn2 immunoassay

Samples	OD_450_
Lcn2 a, 100 ng/ml	1.752 ± 0.123
Lcn2 a, 50 ng/m	0.912 ± 0.165
Lcn2 a, 12.5 ng/m	0.533 ± 0.116
SF9-V	0.294 ± 0.094
Lcn2 a, 50 ng/m	2.501± 0.162
Lcn2 a, 12.5 ng/m	0.095 ± 0.032

a, Standard concentrations of human Lcn2 provided in the ELISA kit

b, 20 – fold dilution

## Discussion

Lcn2 plays important roles in a variety of pathological processes such as intoxication, renal injury, burn injury, human cancers and infection (8, 15-21).

 Lcn2 also acts as a cytoprotective factor against oxidative stress (14). It has been known as the first biochemical marker for some diseases in human. For example, Lcn2 is dramatically up-regulated in the kidney after ischemia (3). Chelation of iron and antibacterial property are other functions of Lcn2 (22). Overall, there is emerging evidences indicating beneficial roles of Lcn2 and its potential clinical application. 

Cloning and expression of Lcn2 have already been reported by other studies. Tong *et al* cloned Lcn2 into expression vector pcDNA3.1 and expressed it in A549 and MCF-7 cell lines (13). Roudkenar *et al* cloned Lcn2 into pcDNA3.1 vector and expressed it in CHO and Hek293T cell lines. In both above mentioned studies, plasmid based vector and mammalian cell lines were used for cloning and expression of the Lcn2 gene (6). 

Considering the probable medical applications of the Lcn2 protein, and also for the purpose of conducting further studies on the biological properties of Lcn2, a large amount of this protein is required. Hence, an expression system with the ability to produce high amounts of this protein is required. One of the popular eukaryotic expression systems is the baculovirus expression vector system (BEVS). BEVS takes advantage of the very late baculoviral promoters; p10 or polyhedrin which are highly transcribed and expressed by infected insect cells. Therefore, considering the high expression yields of this system, and also because of the ease of handling of the insect cells, we evaluated the expression of Lcn2 protein by this system. As far as we know, this is the first report on the expression of the recombinant Lcn2 by a baculovirus expression system and the low cost of the insect cell expression Lcn2 compared to those obtained from mammalian expression systems makes it suitable for further investigations of its concise mechanism and perhaps for the future medical applications.

 To perform the expression procedure, the Lcn2 gene was isolated from HepG2 cell line and transfected into the Sf9 insect cell line through TOPO cloning reaction to investigate the ability of this system to express Lcn2. Here we obtained an expression yield of about 2.5 μg/ml that is much more than that of mammalian expression system.

In a previous study, we expressed Lcn2 in CHO and HEK 293T cell lines (14). In these cells, about 200-250 ng/ml of Lcn2 was produced. Because the mammalian cell lines provide low expression levels, the Sf9 cell line was selected as the “protein factory” for the production of recombinant Lcn2. Insect cells are labeled as an eukaryotic expression system that are not related to some of the defects ascribed to prokaryotic systems (such as the lack of post-translational modifications) and mammalian cell systems (such as low expression levels) (23). On the other hand, baculovirus is one of the most powerful vehicles for foreign gene expression at extremely high levels (24, 25), therefore we used baculovirus as the expression vector system. Another significant point of our work was the employment of both TOPO cloning reaction and gateway system. The gateway system takes advantage of the site specific recombination reactions which eliminates the necessities for the digestion of the gene of interest or plasmid with restriction enzymes (26). 

Furthermore, TOPO cloning is an important asset in projects requiring systematic cloning, modular assembly, and expression in various contexts. Thus, the use of these two methods brings about higher efficiency with simple methodology to produce Lcn2 protein. 

## Conclusion

High-levels of recombinant protein were expressed in SF9 cells. This recombinant Lcn2 could be used to produce monoclonal or polyclonal antibodies that in turn would be used as an early marker for diagnosis of kidney and heart injuries. Recombinant Lcn2 can also be considered in future as a potential therapeutic agent such as antibacterial agent, anti-oxidative factor and iron chelating factor. Large scale expression and purification could be the next steps of our studies.

## References

[1] Devarajan P (2010). Neutrophil gelatinase-associated lipocalin: A promising biomarker for human acute kidney injury. Biomark Med.

[2] Gwira JA, Wei F, Ishibe S, Ueland JM, Barasch J, Cantley LG (2005). Expression of neutrophil gelatinase-associated lipocalin regulates epithelial morphogenesis in vitro. J Biol Chem.

[3] Mori K, Lee HT, Rapoport D, Drexler IR, Foster K, Yang J (2005). Endocytic delivery of lipocalin-siderophoreiron complex rescues the kidney from ischemia-reperfusion injury. J Clin Invest.

[4] Bahmani P, Halabian R, Rouhbakhsh M, Roushandeh AM, Masroori N, Ebrahimi M (2010). Neutrophil Gelatinase-Associated Lipocalin induces the expression of heme oxygenase-1 and superoxide dismutase 1, 2. Cell Stress Chaperones.

[5] Roudkenar MH, Kuwahara Y, Baba T, Roushandeh AM, Ebishima S (2007). Oxidative stress induced lipocalin 2 gene expression: addressing its expression under the harmful conditions. J Radiat Res (Tokyo).

[6] Roudkenar MH, Halabian R, Roushandeh AM, Nourani MR, Masroori N, Ebrahimi M (2009). Lipocalin 2 regulation by thermal stresses: Protective role of Lcn2/NGAL against cold and heat stresses. Exp Cell Res.

[7] Jarmi T, Agarwal A (2009). Heme oxygenase and renal disease. Curr Hypertens Rep.

[8] Zhang H, Xu L, Xiao D, Xie J, Zeng H, Wang Z (2007). Upregulation of neutrophil gelatinase-associated lipocalin in oesophageal squamous cell carcinoma:significant correlation with cell differentiation and tumour invasion. J Clin Pathol.

[9] Holmes MA, Paulsene W, Jide X, Ratledge C, Strong RK (2005). Siderocalin (Lcn 2) Also Binds Carboxymycobactins, Potentially Defending against Mycobacterial Infections through Iron Sequestration. Structure (London, England: 1993).

[10] Schmidt-Ott KM, Mori K, Li JY, Kalandadze A, Cohen DJ, Devarajan P (2007). Dual Action of Neutrophil Gelatinase-Associated Lipocalin. J Am Soc Nephrol.

[11] Breustedt DA, Schönfeld DL, Skerra A (2006). Comparative ligand-binding analysis of ten human lipocalins. Biochim Biophys Acta.

[12] Lee HJ, Lee EK, Lee KJ, Hong SW, Yoon Y, Kim JS (2006). Ectopic expression of neutrophil gelatinase-associated lipocalin suppresses the invasion and liver metastasis of colon cancer cells. Int J Cancer.

[13] Tong Z, Wu X, Ovcharenko D, Zhu J, Chen CS, Kehrer JP (2005). Neutrophilgelatinase-associatedlipocalin as a survivalfactor. Biochem J.

[14] Roudkenar MH, Halabian R, Ghasemipour Z, Roushandeh AM, Rouhbakhsh M, Nekogoftar M (2008). Neutrophil gelatinase-associated lipocalin acts as a protective factor against H(2)O(2) toxicity. Arch Med Res.

[15] Bauer M, Eickhoff JC, Gould MN, Mundhenke C, Maass N, Friedl A (2008). Neutrophil gelatinase-associated lipocalin (NGAL) is a predictor of poor prognosis in human primary breast cancer. Breast Cancer Res Treat.

[16] Cho H, Kim JK (2009). Lipocalin2 expressions correlate significantly with tumor differentiation in epithelial ovarian cancer. J Histochem Cytochem.

[17] Hemdahl AL, Gabrielsen A, Zhu C, Eriksson P, Hedin U, Kastrup J (2006). Expression of neutrophil gelatinase-associated lipocalin in atherosclerosis and myocardial infarction. Arterioscler Thromb Vasc Biol.

[18] Katano M, Okamoto K, Arito M, Kawakami Y, Kurokawa MS, Suematsu N (2009). Implication of granulocyte-macrophage colony-stimulating factor induced neutrophil gelatinase-associated lipocalin in pathogenesis of rheumatoid arthritis revealed by proteome analysis. Arthritis Res Ther.

[19] Mishra J, Mori K, Ma Q, Kelly C, Barasch J, Devarajan P (2004). Neutrophil Gelatinase-Associated Lipocalin: A Novel Early Urinary Biomarker for Cisplatin Nephrotoxicity. Am J Nephrol.

[20] Tong Z, Wu X, Ovcharenko D, Zhu J, Chen CS, Kehrer JP (2005). Neutrophil gelatinase-associated lipocalin as a survival factor. Biochem J.

[21] Vemula M, Berthiaume F, Jayaraman A, Yarmush ML (2004). Expression profiling analysis of the metabolic and inflammatory changes following burn injury in rats. Physiol genomics.

[22] Roudkenar MH, Halabian R, Oodi A, Roushandeh AM, Yaghmaei P, Najar MR (2008). Upregulation of neutrophil gelatinase-associated lipocalin, NGAL/Lcn2,in b-Thalassemia patients. Arch Med Res.

[23] Masroori N, Halabian R, Mohammadipour M, Roushandeh AM, Rouhbakhsh M, Najafabadi AJ (2010). High-level expression of functional recombinant human coagulation factor VII in insect cells. Biotechnol Lett.

[24] Cha HJ, Dalal NG, Pham MQ, Vakharia VN, Rao G, Bentley WE (1999). Insect larval expression process is optimized by generating fusions with green fluorescent protein. Biotechnol Bioeng.

[25] Miller L.K (1989). Insect baculoviruses: Powerful gene expression vectors. Bioessays.

[26] Gholoobi A, Sankian M, Zarif R, Farshadzadeh Z, Youssefi F, Sadeghian A (2010). Molecular cloning, expression and purification of protein TB10.4 secreted by mycobacterium tuberculosis. Iran J Basic Med Sci.

